# Evaluation of Effectiveness of a Toothpaste Containing Tea Tree Oil and Ethanolic Extract of Propolis on the Improvement of Oral Health in Patients Using Removable Partial Dentures

**DOI:** 10.3390/molecules26134071

**Published:** 2021-07-03

**Authors:** Karolina Wiatrak, Tadeusz Morawiec, Rafał Rój, Patryk Kownacki, Aleksandra Nitecka-Buchta, Damian Niedzielski, Piotr Wychowański, Agnieszka Machorowska-Pieniążek, Armand Cholewka, Domenico Baldi, Anna Mertas

**Affiliations:** 1 Department of Oral Surgery, Faculty of Medical Sciences in Zabrze, Medical University of Silesia in Katowice, Pl. Akademicki 17, 41-902 Bytom, Poland; szczepa.karol@gmail.com (K.W.); tmorawiec@sum.edu.pl (T.M.); p.kownacki1975@gmail.com (P.K.); 2 Department of Dental Prosthetics, Faculty of Medical Sciences in Zabrze, Medical University of Silesia in Katowice, Plac Akademicki 17, 41-902 Bytom, Poland; rafstoma@gmail.com; 3 Department of Temporomandibular Disorders, Faculty of Medical Sciences in Zabrze, Medical University of Silesia in Katowice, Traugutta 2, 41-800 Zabrze, Poland; aleksandra.nitecka@sum.edu.pl; 4 Department of Cranio-Maxillo-Facial Surgery, Faculty of Medical Sciences in Zabrze, Medical University of Silesia in Katowice, Francuska 20/24, 40-027 Katowice, Poland; niedzielskidamian@gmail.com; 5 Department of Oral Surgery, Medical University of Warsaw, Nowogrodzka 59, 02-006 Warszawa, Poland; piotrwychowanski@wychowanski.pl; 6 Department of Orthodontics, Faculty of Medical Sciences in Zabrze, Medical University of Silesia in Katowice, Traugutta 2, 41-800 Zabrze, Poland; agamach@onet.pl; 7 Institute of Biomedical Engineering, Faculty of Science and Technology, University of Silesia in Katowice, 75 Pułku Piechoty 1A, 41-500 Chorzów, Poland; armand.cholewka@us.edu.pl; 8 Department of Surgical and Integrated Diagnostics Sciences, University of Genoa, Via Balbi 5, 16126 Genoa, Italy; baldi.domenico@libero.it; 9 Department of Microbiology and Immunology, Faculty of Medical Sciences in Zabrze, Medical University of Silesia in Katowice, Jordana 19, 41-808 Zabrze, Poland

**Keywords:** oral microbiota, oral hygiene, antimicrobial natural products

## Abstract

The aim of this study was to evaluate the effect of toothpaste containing natural tea tree essential oil (TTO) and ethanolic extract of propolis (EEP), on microflora and selected indicators of oral health in patients using removable acrylic partial dentures. Fifty patients with varying conditions of hygiene were divided into two groups. The study group received the toothpaste with TTO and EEP, while the control group received the same toothpaste but without TTO and EEP. At the first visit, oral hygiene and hygiene of the prostheses were carried out. Control visits took place 7 and 28 days later and compared to baseline. Indexes like API (Approximal Plaque Index), mSBI (modified Sulcus Bleeding Index), OHI-s (simplified Oral Hygiene Index), and DPI (Denture Plaque Index) were assessed in three subsequent stages, and swabs were collected from floor of the mouth area to assess the microbiota. After 7 and 28 days of using the toothpaste with TTO and EEP, a statistically significant decrease of the examined indicator values were observed in the study group as compared to the values upon the initial visit. The number of isolated strains of microorganisms in the study group was decreased or maintained at the same level, whereas in the control group an increase in the number of isolated strains was observed. The observed stabilization of oral microbiota in patients from the study group confirms the beneficial activity of toothpaste containing EEP and TTO compared to the control group.

## 1. Introduction

Plant essential oils are frequently associated with and commonly used in aromatherapy. They exhibit relaxing, analgesic, antiphlogistic, and antiseptic activity. This is, however, not the major field in which they are used. More and more frequently, they are employed in medicine and dentistry as alternative therapies. It is mostly their antiseptic effect that is utilized. They are also known for their antioxidant activity and are often used in food preservation, as spasmolytic agents, and in local anaesthesia [1,2].

Essential oils are concentrated compounds extracted from plants, usually with a strong smell, produced by certain plants as secondary metabolites. They can be present in all parts of plants, including buds, flowers, leaves, seeds, branches, stems, fruits, roots, and wood, but they are most frequently stored by the plant in specialized secretory cells. They have lipophilic properties, which enables them to easily penetrate through the stratum corneum, mucous membrane, and the cell membranes of microorganisms. The lipophilicity of essential oils is the basis of their antiseptic properties. Through combination with the cell membranes of microorganisms they increase permeability and thus trigger the flow of intracellular components and enzyme inactivation [1,3]. Essential oils contain a wide range of terpene and terpenoid compounds which may inhibit or prevent the growth of bacteria, yeasts and mold [4,5]. The basic mechanism of microorganism cell death is loss of cellular membrane integrity or function under the influence of terpenes [6].

Essential oils can exhibit multidirectional antifungal effects, particularly against the *Candida* species, when they inhibit ergosterol synthesis [7], modify cell wall morphology [8], inhibit enzymes involved in cell wall synthesis [8,9], modify the permeability of the cell membrane [10,11] and produce reactive oxygen species [12]. Antifungal activity is extremely important in patients undergoing prosthetic treatment [13]. The oral cavity is a natural habitat of microflora and *Candida* spp. are commensal microorganisms, which may colonize the oral cavity and are usually located on the posterior part of the tongue and on the oral mucosa [14]. The individual composition of the flora is influenced by, inter alia: age, oral cavity topography, condition of dentition, nutritional and breathing habits, tobacco smoking, oral hygiene, loss of teeth, and usage of prosthetic restorations. Acrylic base of partial dentures covers a substantial portion of the surface of the mucosa, creating favorable conditions for the accumulation of bacteria and fungi in the form of denture plaque. It is a place characterized by high humidity, elevated temperature, reduced oxygen supply, and worse conditions for salivary self-cleaning. Moreover, the acrylic material, having a heterogeneous and porous structure, absorbs water and swells in the oral cavity, further facilitating the aggregation of microorganisms [13,15,16].

Extensive use of antibiotics leads to increasing drug resistance of microorganisms. It is worth noting that access to antifungal medication is limited in comparison with antibiotics [17], which, combined with the increased drug resistance, particularly within biofilms, has led to a larger interest in discovering new antimicrobial agents, especially fungicides [18]. Essential oils, as natural antimicrobial substances, are commonly used in dentistry. Clove oil is among the most popular. It contains, inter alia, eugenol (the main component), isoeugenol, acetyleugenol, and terpenes [19,20,21]. Eugenol is an ingredient of root canal sealers, mummifying pastes, zinc oxide eugenol and surgical cements, preparations used in periodontal diseases, and herbal preparations for mouth and throat rinsing [22]. Another popular essential oil is eucalyptus oil. The main active ingredient responsible for its properties is 1,8-cineole, classified as a terpene compound commonly referred to as eucalyptol [23]. Eucalyptus oil has antimicrobial, antifungal, antioxidant, and antiphlogistic effects [2]. Cinnamon oil is also used in dentistry as it inhibits the growth of bacteria and fungi. Research has proven the antifungal effect of cinnamon oil in acrylic denture wearers without a destructive effect on the acrylic material [24]. Lavender oil contains a wide range of terpenoid compounds. Among numerous components of the mixture of chemical compounds, namely distillates and extracts, the dominant ones are linalyl acetate and linalool [25]. The popularity of lavender oil is due to its olfactory characteristics applied in aromatherapy, its healing properties, and a wide range of biological activities also used in treatment of oral diseases [26,27]. Other popular essential oils include lemon oil and peppermint oil [28].

Tea tree oil (*Melaleuca alternifolia*) is one of the most potent plant antiseptics. It is very popular in dermatology and cosmetology. Tea tree oil easily penetrates through external skin layers due to such properties as lipophilicity and high solubility in the secretion of sebaceous glands [29,30,31]. It is used to treat skin disorders caused by fungal infections such as candidiasis, viral infections such as herpes, bacterial infections such as acne vulgaris, dandruff, frostbites, burns, ulcers, and psoriasis. In dentistry, it is used in the treatment of oral candidiasis, angular cheilitis, and prosthetic stomatopathy, i.e., a group of infections caused by *Candida* spp. [32,33,34,35,36]. The risk of candidiasis is higher among patients with lowered immunity, after antibiotic therapy, using acrylic or other dentures, patients with reduced salivary flow, and in the elderly. It is especially among the elderly that all these factors frequently coexist. Furthermore, the use of infected dentures may cause local infection of the oral mucosa as well as respiratory tract or digestive tract infections [37,38]. That is why it appears advisable for wearers of partial functionally unstable dentures to use toothpastes containing tea tree oil. The next antiseptic ingredient used in the studied toothpaste is the ethanolic extract of propolis, which is prepared by extracting crude propolis using 70% ethanol [39]. In dentistry, propolis is used in the treatment of caries and periodontal diseases; it also exhibits antiphlogistic and antifungal activities [40,41,42].

The aim of this study was to evaluate the influence of toothpaste including in its composition natural antimicrobial products such as ethanolic extract of propolis (EEP) and tea tree oil (TTO), on the oral microbiota, and to calculate the values of the selected oral health indices (approximal plaque index—API; modified sulcus bleeding index—mSBI; simplified oral hygiene index—OHI-s; denture plaque index—DPI) in patients using partial acrylic removable dentures.

## 2. Results

### 2.1. Oral Health Conditions

#### 2.1.1. The Approximal Plaque Index (API)

**Figure 1:**
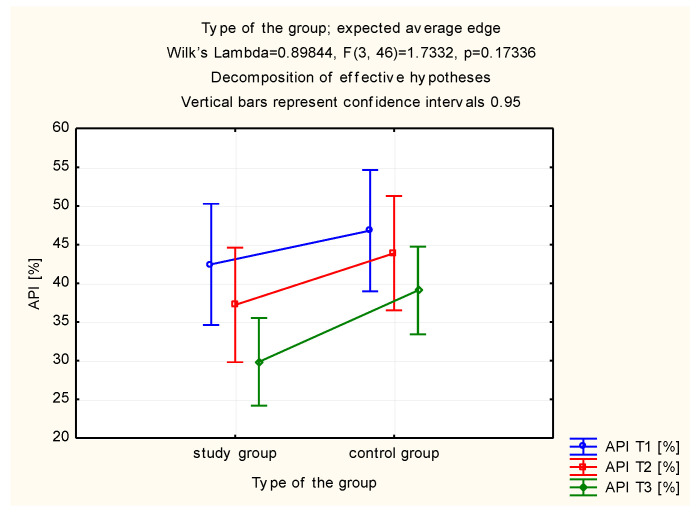
Estimated marginal means for the approximal plaque index (API) in the study and control groups.

The general range of distribution for API was presented in Table 1. Statistically significant differences between studied groups of patients (the study group and the control group were in detail described in chapter 4.1.) were not reported 7 and 28 days after the initial examination (*p* < 0.39 and *p* < 0.38). However, a clear difference in the improvement of the API between the first and the last visit was noted between the study (A) and control (C) groups (*p* < 0.002 vs. *p* < 0.03). Improvement of oral hygiene was reported both in the study and in the control groups. This improvement may be associated with the reduction of interproximal spaces in the lateral segments of the maxilla and/or mandible. A significant improvement was observed only in the study group (Table 1, Figure 1).

#### 2.1.2. The Simplified Oral Hygiene Index (OHI-s)

**Figure 2:**
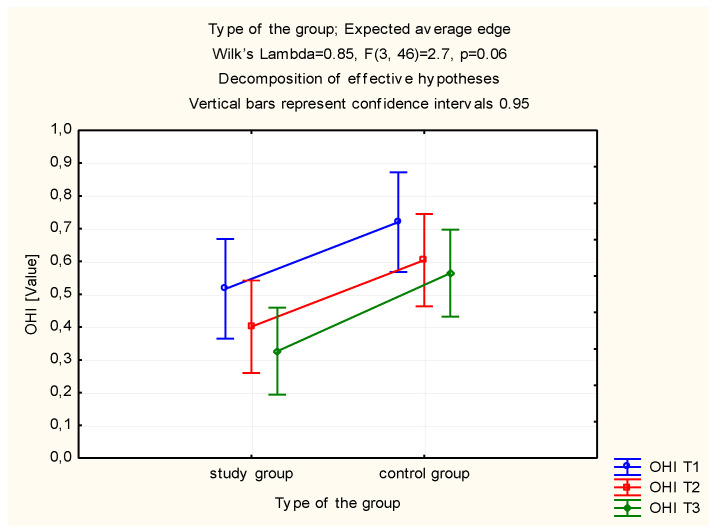
Estimated marginal means for the simplified oral hygiene index (OHI-s) in the study and control groups.

Another assessed index was the simplified Greene–Vermilion hygiene index. Statistically significant improvement of hygiene in the study group was observed (Table 2). In this group, OHI-s proved to be statistically significantly higher at the beginning of the period of using the toothpastes than after 28 days of usage (*p* = 0.049). No statistical significance was observed in the control group (Table 2). Significant differences were observed by comparing the OHI-s at 7 and 28 days after the initial examination between the study (A) group and the control (C) group (*p* = 0.046, *p* = 0.014) (Table 2, Figure 2).

#### 2.1.3. The Denture Plaque Index—DPI (Budtz-Jørgensen)

**Figure 3:**
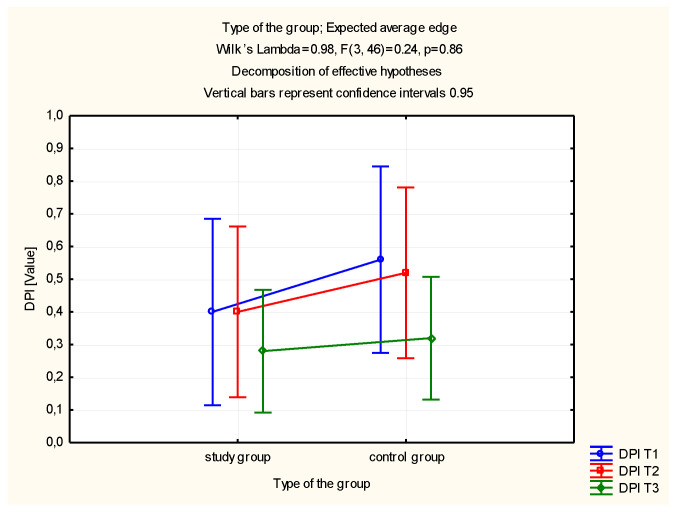
Estimated marginal means for the denture plaque index (DPI) in the study and control groups.

Based on the conducted analysis of denture plaque index values, no significant reduction in the values was observed after the period of use of the toothpastes in the study and control groups, as compared to the output value (Table 3, Figure 3).

#### 2.1.4. The modified Sulcus Bleeding Index (mSBI)

**Figure 4:**
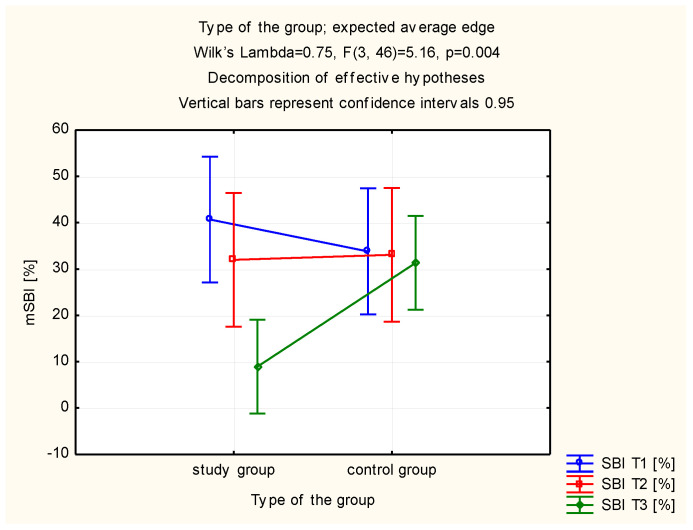
Estimated marginal means for the modified sulcus bleeding index (mSBI) in the study and control groups.

A statistically significant decrease of the SBI value was observed in the study group, after 28 days of use of the toothpaste with TTO and EEP, compared to the SBI values observed at the initial visit (Table 4). The study and control groups did not differ in terms of the assessment of the SBI value upon initial examination. After 7 and 28 days of use (*p* = 0.727 vs. *p* = 0.627 vs. *p* = 0.118) the control group exhibited a significantly higher level of SBI in comparison with the study group (Table 4, Figure 4).

### 2.2. Microbiological Investigation

**Figure 5:**
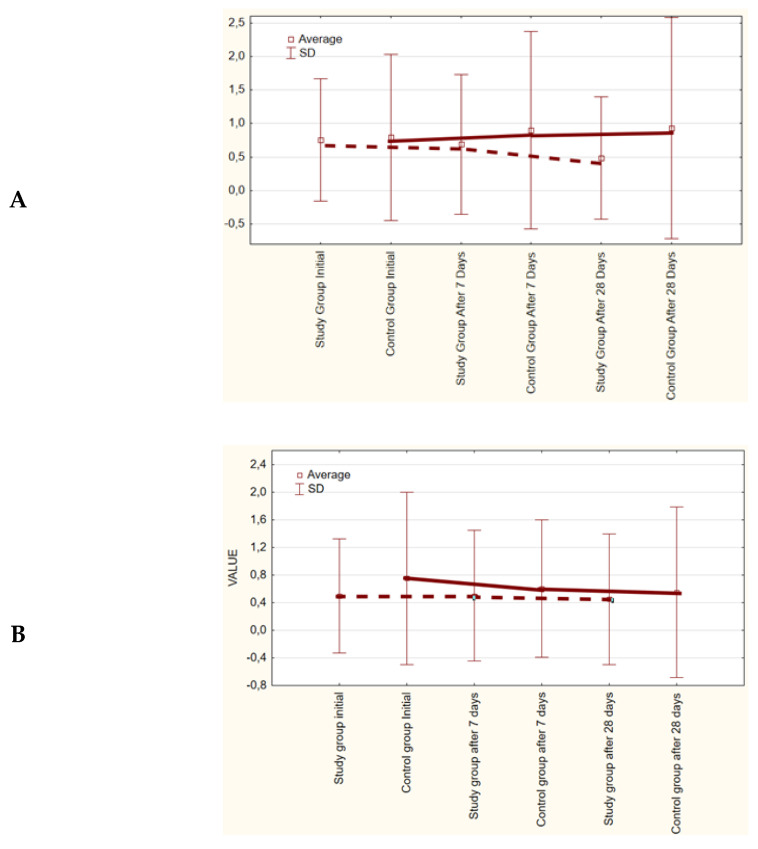

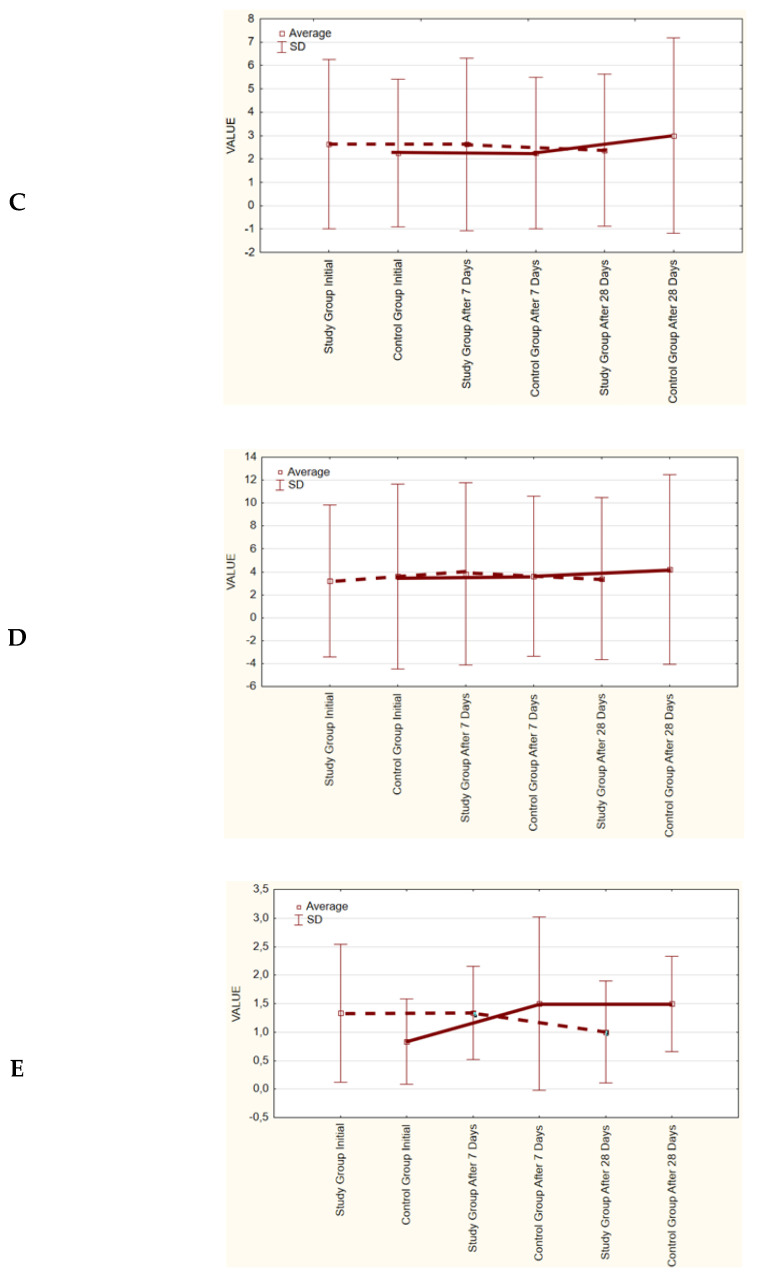
Estimated marginal means for microorganism strains in the study and control groups (initial, after 7 days, and after 28 days): (**A**)—Gram(+) anaerobic bacteria; (**B**)—Gram(-) anaerobic bacteria; (**C**)—Gram(+) aerobic bacteria; (**D**)—Gram(−) aerobic bacteria; (**E**)—fungi.

The total number of bacterial and fungal strains isolated from smears collected from patients in the study group decreased from 108 microorganism strains on the first day of the study to 96 strains after 7 days, and then to 80 strains after 28 days of using the toothpaste with TTO and EEP. The number of isolated microorganism strains in the control group increased from 100 strains isolated on the first day of the study to 101 strains isolated after 7 days and 104 strains after 28 days. A decrease in the number of isolated microorganism strains was observed in all groups of bacteria species observed in the smears from the study group (Table 5, Figure 5). The number of isolated strains of Gram-positive anaerobes was reduced from 30 to 15 strains, in the case of Gram-positive aerobes from 29 to 25 isolated strains, as regards Gram-negative anaerobes from 16 to 13 strains and Gram-negative aerobes from 24 to 21 microorganism strains, considering the first and the final examinations. In the control group, the number of isolated anaerobic bacterial strains decreased. The number of isolated Gram-positive anaerobes decreased from 25 to 23 strains, and the number of isolated Gram-negative anaerobes from 19 to 17 strains. However, the number of isolated aerobic bacterial strains increased: Gram-positive bacterial strains increased from 26 to 29 strains and Gram-negative bacterial strains increased from 22 to 26 strains, considering the first and the final examinations. The number of isolated *Candida albicans* strains decreased from 9 to 6 in the study group and increased from 8 to 9 strains in the control group. Apart from quantitative changes, qualitative variation of the oral microbiota was also observed, as presented in detail in Table 6.

## 3. Discussion

Nowadays, for prophylaxis and for the treatment of numerous conditions, we return more often to natural substances showing antimicrobial properties. More often they appear as substances of plant origin with complex composition and, frequently, multidirectional effects which suppress the generation of the resistance mechanisms of the microorganisms. Some representative examples of such substances may be propolis and tea tree oil (TTO). Tea tree oil is a well-known antiseptic with antimicrobial, antiviral, and antifungal effects. As mentioned in the introduction, its antiseptic properties are due to terpene compounds, which can be used in dentistry to maintain proper oral hygiene with toothpastes and rinses, prevent caries, and to treat mucosal diseases and candidiasis [33,43,44,45]. Another antimicrobial natural substance used in the studied toothpaste was ethanolic extract of propolis. Crude propolis is very rarely used for medical or industrial purposes. Its most common form is its ethanolic extract which, throughout its extraction, is purified of ballast substances and mechanical additives [46]. Ethanolic extract of propolis was used due to its antiphlogistic and antiseptic properties [47]. It should be remembered that propolis extracts may be allergenic when in contact with the skin or mucous membranes, which is a type 4 delayed allergic reaction [17]. Also, TTO may cause allergic reactions, especially in predisposed individuals. The dose-dependent toxicity of TTO can be avoided by using this essential oil in a diluted form [43].

This study demonstrated a decrease in the number of isolated bacterial strains and fungi in patients with removable partial dentures using toothpaste with EEP and TTO in comparison with the control group, which allows for the conclusion that the substances used in the toothpaste exhibit antimicrobial and antifungal activity. We previously studied the toothpaste with only ethanolic extract of propolis as antimicrobial substance [42]. We confirmed the antimicrobial activity of a gel with propolis in patients who underwent implant-supported prosthodontic rehabilitation [47], as well as in patients after tooth extraction procedures, which directly influenced better healing in these groups of patients [48]. Feres et al. [49] compared the antimicrobial activity of 11% propolis and the popular 0.12% chlorhexidine against bacteria in the saliva of healthy patients and patients with periodontal diseases. Both substances exhibited statistically significant antimicrobial activities. A 2013 study also compared 0.12% chlorhexidine with 2% propolis. Its antimicrobial activity against *Streptococcus mutans* and *Lactobacillus acidofilus* was likewise confirmed [50]. A study on the efficacy of toothpastes with natural extracts against three species of bacteria *Streptococcus mutans, Pseudomonas aeruginosa* and *Enterococcus faecalis* compared to a toothpaste without antimicrobial ingredients was conducted in 2015. The results demonstrated that *Enterococcus faecalis* exhibited resistance to the toothpaste with propolis and tea tree oil and susceptibility to the toothpaste with propolis only. According to the authors, this was due to a different concentration of propolis in the toothpastes. Propolis/tea tree oil toothpaste exhibited the strongest antimicrobial activity against *Streptococcus mutans* compared to other toothpastes. *P. aeruginosa* proved to be resistant to all tested natural substances [51]. In the present study, we evaluated a toothpaste with more ingredients than only ethanolic extract of propolis. The enrichment of the composition of the toothpaste with the addition of tea tree oil increased its effectiveness and antimicrobial activity over the control toothpaste without antimicrobial substances. After 28 days of use of the toothpaste with EEP and TTO, oral microbiota diversity was more reduced than in the control group (Table 6): 15 microbial species were eliminated (13 species in the control group), 21 microbial species declined (9 species in the control group), 9 microbial species were gained (10 species in control group), and only 5 microbial species were increased (up to 9 species in the control group). This study confirmed the antimicrobial properties of traditional medicinal substances like essential oils against oral microbiota, especially against oral pathogens, and suggests they could be beneficial in caries, periodontal disease prevention, endodontics, and candidiasis treatment [52,53,54,55,56].

This study succeeded in demonstrating a statistically significant reduction of the modified sulcus bleeding index (mSBI) as modified by the authors in the group using the toothpaste with EEP and TTO. Moreover, gingival bleeding on probing was present in 52% of study group patients at the beginning of the study. After 28 days of using the toothpaste, none of these patients showed bleeding on probing. This may be evidence of the beneficial effects of the studied substances on the periodontium and confirms their antiphlogistic properties. The 2017 study of oral rinse with TTO compared to 0.12% chlorhexidine and placebo demonstrated a decrease in gingivitis indices such as GI and FMBS in patients using the TTO rinse. Propolis also exhibits antiphlogistic activity, which was demonstrated by Barroso et al. [57]. In their study, the antiphlogistic activity of propolis, in which mast cells participate, was more effective than that of dexamethasone in the inflammatory phase of healing. In present study, there was a decrease in the number of isolated *Candida albicans* strains in patients using the active toothpaste, whereas patients using placebo demonstrated an increase in the number of *Candida albicans* colonies cultured. In both groups, the differences were not statistically significant. Both active substances employed in the active toothpaste exhibit antifungal activity. However, it should be remembered that the examined patients were using acrylic partial functionally unstable dentures, which are an iatrogenic factor for *Candida albicans* infection. The use of acrylic partial functionally unstable dentures causes changes in the oral microbiota. The acrylic material is a reservoir for *Candida albicans*, the quantity of which depends on denture hygiene and quality, and particularly on roughness [15,16,58].

## 4. Materials and Methods

### 4.1. Studied Groups of Patients

The study included a total 50 patients using removable partial dentures, aged 41–82 years (61.08 ± 12.49), including 28 females and 22 males. Patients were randomly divided into two groups: the study group consisted of 25 patients aged 41–79 years (55.05 ± 11.09), whereas the control group consisted of 25 patients aged 41–82 years (66.79 ± 11.19). Patients from the study group used a toothpaste with ethanolic extract of propolis and tea tree oil with the following qualitative composition: aqua (up to 100% of weight), glycerine (5–12%), silica (10–14%), sorbitol (10–20%), hydroxyethyl cellulose (0.1–1%), titanium dioxide (0.5–2%), xanthan gum (0.3–1%), ethanolic extract of propolis (EEP) (1.0%), tea tree oil (TTO) (1.0%), menthol oil (0.2%), and rosemary oil (0.1%). While patients from the control group used the so-called placebo toothpaste, containing toothpaste base with abrasives, but without active substances. The qualitative composition of the control (placebo) toothpaste: aqua, glycerine, silica, sorbitol, hydroxyethyl cellulose, titanium dioxide, xanthan gum. The AS Cosmetics Sernice (Warsaw, Poland) and Melaleuca Poland Sp. z o.o. (Gliwice, Poland) companies were responsible for the composition and production of the toothpastes. Australian tea tree oil from *Melaleuca alternifolia*, fulfilling the ISO 4730:2004 requirements was used in the active toothpaste, together with Polish propolis produced by *Apis mellifera* bees from apiaries located in northwestern Poland, fulfilling the Polish Standard for Propolis Concentrate PN-A-77627.

After receiving patient consent for participation in the study, general medical and dental interviews were conducted. Patients received the active or control toothpaste and were instructed to brush their teeth twice a day at least for two minutes and to refrain from using any other oral care agents until the end of the experiment.

### 4.2. Inclusion and Exclusion Criteria

Inclusion criteria: 40–85 years of age, written participation consent, patients lacking 5 to 8 teeth in the maxilla and mandible with planning removable dentures, patients with remaining posterior occlusal pairs with a minimum of two supporting zones according to the Eichner index. Exclusion criteria: lack of written participation consent, painful masticatory dysfunction, edentulous patients, or patients with residual dentition (Eichner subgroups C1–C3), patients with full dental arches or lacking 1–2 teeth in an arch, patients with retaining teeth mobility over +20 PTV tested with the Periotest instrument, patients with class V fillings, acrylic/ceramic crowns around the retaining teeth, patients with cancer, psychosomatic disorders, patients after trauma within the craniofacial region, pregnant and lactating females, patients suffering from asthma, atopic dermatitis, allergy to foods, drugs, honey and other bee products or having other allergy-related ailments. The study was conducted with prior approval from the Bioethics Committee of the Silesian Medical Chamber in Katowice, Poland, Resolution No 8/2015 of 23 March 2015.

### 4.3. Indices

Persons qualified for the study were evaluated to determine the value of the selected indices, as modified by the authors, i.e., mSBI, API, OHI-S, DPI. In order to obtain a full dental status and detect any latent inflammatory foci, a pantomographic image was routinely taken. The approximal plaque index (API) according to Lange, and as modified by the authors is used to evaluate the efficiency of conducted hygienic treatments. The index was adjusted to the studied group of patients with edentulism. The presence of plaque was evaluated only on the occlusal surfaces of adjacent teeth. Lack of an adjacent tooth meant the lack of an occlusal surface for evaluation. The modified sulcus bleeding index (mSBI), as modified by the authors, enables the evaluation of the severity of the inflammatory response within periodontal tissues, taking into consideration only the presence (+) or absence (-) of bleeding. The index describes the presence of localized bleeding in interdental spaces and in the interdental papilla. Due to edentulism in the studied group of patients, the original mSBI was modified by the authors. When evaluating the index, only the interdental spaces were considered. Lack of an adjacent tooth rendered evaluation impossible. The simplified oral hygiene index (OHI-s) according to Greene and Vermillion, as modified by the authors, is based on the evaluation criteria of the debris index (DI) and the calculus index (CI). The amount of deposit on six surfaces of six teeth was evaluated. The last evaluation was the denture plaque index (DPI).

### 4.4. Material for Microbiological Examination

A smear from the floor of the mouth was collected from each of the patients. Smears were collected with use of sterile swabs and placed in a transport medium, then delivered to the Microbiological Laboratory at the Department of Microbiology and Immunology in Zabrze, where microbiological tests were conducted. Both groups were instructed on how to perform proper oral hygiene and denture care. Control visits took place 7 and 28 days after the first visit. Control visits consisted of evaluation of the API and mSBI (as modified by the authors) and microbiological material was collected. Patients were examined for regression of lesions, frame of mind, and possible side effects.

### 4.5. Microbiological Examinations

Microbiological examinations were performed with the use of classic methods employed in laboratory microbiological diagnostics. Material collected from the patients was delivered to the laboratory on the day of collection and immediately cultured in growth media for proliferation and isolation of pure cultures. Aerobic bacteria were proliferated on Columbia Agar solid medium with 5% sheep blood at 37 °C. Anaerobic bacteria were proliferated on Schaedler K3 solid medium with 5% sheep blood at 37 °C in anaerobic conditions obtained with Biomerieux GENbag anaer generators (Marcy l’Étoile, France). Fungi of the *Candida* genus were proliferated and initially identified with use of ChromID Candida chromogenic medium (Biomerieux, Marcy l’Étoile, France). After isolation and proliferation of cultured microorganism strains, their species were identified with use of the following reagent kits: ENTEROtest 24 N, NEFERMtest 24 N, STREPTOtest 24, STAPHYtest 24, ANAEROtest 23, OXItest; the PYRAtest and the TNW_lite 6.5 computer program were used for species identification of microorganisms (Erba-Lachema, Brno, Czech Republic). Also, the following Biomerieux (Marcyl’Étoile, France) biochemical tests were used: Katalaza, Slidex Staph Kit, API Candida. Performance and interpretation of results of the tests were carried out according to the manufacturer’s recommendations with diagnostic reagent kits.

### 4.6. Statistical Analysis

The first stage of statistical analysis consisted of the verification of the compatibility of the obtained index values and the number of bacteria with normal distribution with the use of the Shapiro–Wilk test. Variables with normal distribution were presented with arithmetic mean and standard deviation. Nonparametric variables were presented with median and interquartile range. One-way analysis of variance ANOVA and Levene’s test were used to compare the results of the study group with the control group for OHI, the denture plaque index, and the number of bacteria. The comparison of results between groups was performed with the Tukey–Kramer method. The results obtained for OHI and the denture plaque index were compared using the Student’s *t*-test for dependent and independent samples. For unrelated variables of API and mSBI, the results of the study and control groups were compared using the Mann–Whitney U test, the Wilcoxon signed-rank test and Friedman’s ANOVA with Kendall’s coefficient of concordance. The results were deemed to be statistically significant if *p* < 0.05.

## 5. Conclusions

The beneficial effect of the studied toothpaste containing ethanolic extract of propolis (EEP) and tea tree oil (TTO) on oral hygiene and periodontal condition was observed in the study group. Statistically significant reduction of the modified sulcus bleeding index (mSBI) values confirms not only a beneficial impact on the periodontium, but also the antiphlogistic activity of this toothpaste. The use of a toothpaste containing used natural antimicrobial substances significantly influenced the quantitative reduction of oral microbiota, which confirms the antimicrobial and antifungal activity of the used natural antimicrobial substances, such as TTO and EEP. The potential industrial uses of presented results can, in the future, enrich the offer of available oral hygiene products particularly beneficial for oral health.

## Figures and Tables

**Table 1:** Approximal plaque index (API) ranges—assessment for study and control groups.

Oral Hygiene Assessment (Interproximal Spaces)						
	Assessment Criteria	T1 (1)	T2 (2)	T3 (3)	Friedman’s ANOVA Test (*p*)/Kendall’s Coefficient of Concordance	Wilcoxon Signed-Rank Test (*p*)
Study Group (A)	Optimal	12% (3 persons)	44% (11 persons)	60% (15 persons)	*p* = 0.00001/0.46	(1):(2) = 0.00114 (2):(3) = 0.088 (1):(3) = 0.0019
	Quite good	20% (5 persons)	32% (8 persons)	36% (9 persons)		
	Average	48% (12 persons)	24% (6 persons)	4% (1 person)		
	Bad	20% (5 persons)	0%	0%		
Control Group (C)	Optimal	16% (4 persons)	24% (6 persons)	40% (10 persons)	*p* = 0.008/0.192	(1):(2) = 0.257 (2):(3) = 0.066 (1):(3) = 0.028
	Quite good	12% (3 persons)	36% (9 persons)	40% (10 persons)		
	Average	68% (17 persons)	40% (10 persons)	20% (5 persons)		
	Bad	4% (1 person)	0%	0%		
Mann-Whitney U test (*p*)		0.515	0.388	0.382	-	-

T1—preliminary examination before hygienic procedure; T2—examination after 7 days; T3—28 days after the initial examination.

**Table 2:** Simplified oral hygiene index (OHI) ranges—assessment for study and control groups.

Oral Hygiene Assessment (Interproximal Spaces)											
	T1		Mean ± Standard Deviation	T2		Mean ± Standard Deviation	*p*	T3		Mean ± Standard Deviation	*p*
Study Group (A)	0–0.5	60% (15 persons)	0.52 ± 0.24	0–0.5	68% (17 persons)	0.40 ± 0.33	0.14	0–0.5	80% (20 persons)	0.33 ± 0.39	0.049
	0.6–1	40% (10 persons)		0.6–1	32% (8 persons)			0.6–1	20% (5 persons)		
	1.1–2	0%		1.1–2	0%			1.1–2	0%		
	2.1–3	0%		2.1–3	0%			2.1–3	0%		
Control Group (C)	0–0.5	56% (14 persons)	0.72 ± 0.48	0–0.5	64% (16 persons)	0.60 ± 0.37	0.179	0–0.5	64% (16 persons)	0.57 ± 0.23	0.076
	0.6–1	24% (6 persons)		0.6–1	24% (6 persons)			0.6–1	35% (9 persons)		
	1.1–2	16% (4 persons)		1.1–2	12% (3 persons)			1.1–2	0%		
	2.1–3	5% (1 person)		2.1–3	0%			2.1–3	0%		
Study (A) vs. Control (C) (*p*)	0.063			0.046				0.014			

T1—preliminary examination before hygienic procedure; T2—examination after 7 days; T3—28 days after the initial examination.

**Table 3:** Denture plaque index (DPI) ranges—assessment for study and control groups.

Denture Plaque Index (Budtz-Jørgensen)											
	T1		Mean ± Standard Deviation	T2		Mean ± Standard Deviation	*p*	T3		Mean ± Standard Deviation	*p*
Study Group (A)	0	64% (16 persons)	0.41 ± 0.58	0	64% (16 persons)	0.40 ± 0.58	-	0	72% (18 persons)	0.28 ± 0.46	0.083
	1	32% (8 persons)		1	32% (8 persons)			1	28% (7 persons)		
	2	5% (1 person)		2	5% (1 person)			2	0%		
	3	0%		3	0%			3	0%		
Control Group (C)	0	60% (15 persons)	0.56 ± 0.82	0	60% (15 persons)	0.52 ± 0.72	0.574	0	68% (17 persons)	0.32 ± 0.47	0.083
	1	28% (7 persons)		1	28% (7 persons)			1	32% (8 persons)		
	2	8% (2 persons)		2	12% (3 persons)			2	0%		
	3	5% (1 person)		3	0%			3	0%		
Study (A) vs. Control (C) (*p*)	0.43			0.52				0.76			

T1—preliminary examination before hygienic procedure; T2—examination after 7 days; T3—28 days after the initial examination.

**Table 4:** Modified sulcus bleeding index (mSBI) ranges—assessment for study and control groups.

Sulcus Bleeding Index Assessment						
	Assessment Criteria	T1 (1)	T2 (2)	T3 (3)	Friedman’s ANOVA Test (*p*)/Kendall’s Coefficient of Concordance	Wilcoxon Signed-Rank Test (*p*)
Study Group (A)	Normal gingiva SBI < 10%	48% (12 persons)	68% (17 persons)	100% (25 persons)	*p* = 0.27/0.052	(1):(2) = 0.135923 (2):(3) = 0.485171 (1):(3) = 0.005724
Bleeding on probing	52% (13 persons)	32% (8 persons)	0%			
Control Group (C)	Normal gingiva SBI < 10%	56% (14 persons)	64% (16 persons)	72% (18 persons)	*p* = 0.265/0.53	(1):(2) = 0.388187 (2):(3) = 0.610121 (1):(3) = 0.754753
Bleeding on probing	44% (11 persons)	36% (9 persons)	28% (7 persons)			
Mann-Whitney U test (*p*)		0.727	0.627	0.118	-	-

T1—preliminary examination before hygienic procedure; T2—examination after 7 days; T3—28 days after the initial examination.

**Table 5:** Microorganism species isolated from patients using the toothpaste with EEP and TTO (study group) or without EEP and TTO (control group).

Isolated Microorganisms	Number of Patients from Study Group			Number of Patients from Control Group		
	Initial	After 7 Days	After 28 Days	Initial	After 7 Days	After 28 Days
Gram(+) anaerobic bacteria						
*Actinomyces viscocus*	1	1	0	0	1	1
*Actinomyces israelii*	3	2	1	4	2	6
*Actinomyces naeslundii*	3	3	2	5	5	6
*Anaerococcus prevotti*	0	0	1	0	0	0
*Atopobium minutum*	1	0	0	1	0	0
*Atopobium parvulum*	1	0	0	0	0	0
*Bifidobacterium adolesccentis*	3	0	1	1	0	1
*Bifidobacterium dentium*	5	4	3	0	3	2
*Bifidobacterium longum*	0	1	1	0	0	0
*Bifidobacterium breve*	2	2	0	4	3	1
*Bifidobacterium ovatus*	1	0	1	0	0	0
*Blautia producta*	2	1	2	1	0	1
*Clostridium barati*	1	1	0	0	1	0
*Clostridium botulinum biovar A*	0	0	1	0	0	0
*Clostridium butyricum*	0	1	0	0	0	0
*Clostridium clostridiforme*	1	0	0	1	2	0
*Clostridium histolyticum*	0	1	0	0	0	0
*Clostridium perfringens*	0	1	0	0	1	3
*Clostridium sporogenes*	1	1	0	0	0	0
*Gemella morbillorum*	1	0	0	0	0	0
*Lactobacillus acidophilus*	2	1	1	4	2	1
*Lactobacillus catenoformis*	0	0	0	1	0	0
*Lactobacillus fermentum*	1	1	0	1	1	0
*Leuconostoc* spp.	0	0	0	1	0	0
*Propionibacterium propionicum*	0	0	1	1	0	0
*Peptococcus niger*	0	0	0	0	0	1
*Pseudoflavonifractor capillosus*	1	0	0	0	0	0
Gram(−) anaerobic bacteria						
*Bacteroides ovatus*	1	0	0	1	1	0
*Bacteroides uniformis*	1	0	0	0	0	0
*Campylobacter gracilis*	0	2	1	2	1	0
*Capnocytophaga ochracea*	0	1	0	2	1	1
*Mitsuokella multacida*	5	4	8	4	5	7
*Parabacteroides distasonis*	1	1	0	0	1	0
*Prevotella melaninogenica*	0	0	1	0	0	0
*Prevotella oralis*	0	0	0	1	1	0
*Veillonella parvula*	1	1	0	1	2	2
Gram(+) aerobic bacteria						
*Micrococcus* spp.	0	0	1	0	0	0
*Staphylococcus aureus MSSA*	0	0	0	1	0	0
*Staphylococcus epidermidis MSCNS*	1	0	0	0	0	0
*Staphylococcus lentus*	0	0	0	0	0	1
*Streptococcus acidominimus*	0	1	0	0	0	0
*Streptococcus mitis*	10	15	10	12	11	12
*Streptococcus oralis*	0	1	1	0	0	0
*Streptococcus mutans*	0	0	0	0	0	1
*Streptococcus salivarius*	14	9	10	10	12	11
*Streptococcus sanguinis*	4	6	3	3	3	4
Gram(−) aerobic bacteria						
*Acinetobacter baumannii*	1	0	0	0	0	0
*Burkholderia cepacia*	0	0	0	2	0	2
*Citrobacter freundii*	0	0	0	0	1	0
*Enterobaeter amniogenus*	1	1	0	0	0	0
*Enterobacter cloacae*	0	1	0	1	0	0
*Enterobacter kobei*	2	1	1	2	1	0
*Enterobacter* spp.	0	0	0	0	1	1
*Escherichia coli*	1	2	1	1	2	4
*Klebsiella oxytoca*	0	0	0	0	1	2
*Klebsiella pneumoniae*	1	0	0	2	1	0
*Neisseria sicca*	2	0	0	1	3	0
*Neisseria subflava*	22	21	20	20	21	24
*Providencia rustigiani*	0	0	1	0	0	0
*Pseudomonas aeruginosa*	0	0	0	1	1	0
*Pseudomonas* spp.	0	0	0	0	1	0
*Stenotrophomonas maltophilia*	0	0	1	0	0	0
*Serratia plymuthica*	1	0	0	0	0	0
Fungi						
*Candida albicans*	9	8	6	8	9	9
Number of microorganisms strains	108	96	80	100	101	104
Altogether:	284			305		

**Table 6:** Changes of oral microbiota in patients using the toothpaste with EEP and TTO (study group) or without EEP and TTO (control group).

Changes of Microorganism Species after 28 Days of the Study	Study Group	Control Group
Eliminated species	*Actinomyces viscocus* *Atopobium minutum* *Atopobium parvulum* *Bifidobacterium breve* *Clostridium barati* *Clostridium butyricum* *Clostridium histolyticum* *Clostridium perfringens* *Clostridium sporogenes* *Gemella morbillorum* *Serratia plymuthica* *Veillonella parvula* *Klebsiella pneumoniae* *Staphylococcus epidermidis MSCNS* *Neisseria sicca*	*Atopobium minutum* *Clostridium clostridiforme* *Lactobacillus catenoformis* *Leuconostoc* spp. *Propionibacterium propionicum* *Burkholderia cepacia* *Enterobacter kobei* *Klebsiella pneumoniae* *Prevotella oralis* *Pseudomonas aeruginosa* *Staphylococcus aureus MSSA* *Neisseria sicca* *Enterobacter cloacae*
Declined species	*Actinomyces israelii* *Actinomyces naeslundii* *Bifidobacterium dentium* *Clostridium clostridiforme* *Lactobacillus acidophilus* *Lactobacillus fermentum* *Pseudoflavonifractor capillosus* *Acinetobacter baumannii* *Bacteroides ovatus* *Bacteroides uniformis* *Capnocytophaga ochracea* *Enterobacter kobei* *Escherichia coli* *Parabacteroides distasonis* *Streptococcus acidominimus* *Streptococcus mitis* *Streptococcus salivarius* *Streptococcus sanguinis* *Enterobacter cloacae* *Neisseria subflava* *Candida albicans*	*Staphylococcus aureus MSSA* *Neisseria sicca* *Enterobacter cloacae* *Acinetobacter freundii* *Bacteroides ovatus* *Campylobacter gracilis* *Capnocytophaga ochracea* *Parabacteroides distasonis* *Pseudomonas* spp.
Gained species	*Anaerococcus prevotti* *Bifidobacterium longum* *Blautia producta* *Propionibacterium propionicum* *Prevotella melaninogenica* *Stenotrophomonas maltophilia* *Micrococcus* spp. *Streptococcus oralis* *Providencia rustigiani*	*Actinomyces viscocus* *Blautia producta* *Peptococcus niger* *Enterobacter* spp. *Veillonella parvula* *Streptococcus mitis* *Streptococcus mutans* *Streptococcus salivarius* *Streptococcus sanguinis* *Candida albicans*
Increased species	*Bifidobacterium ovatus* *Clostridium botulinum biovar A* *Campylobacter gracilis* *Enterobacter amniogenus* *Mitsuokella multacida*	*Actinomyces israelii* *Actinomyces naeslundii* *Clostridium perfringens* *Escherichia coli* *Klebsiella oxytoca* *Mitsuokella multacida* *Staphylococcus lentus* *Burkholderia cepacia* *Neisseria subflava*

## Data Availability

Department of Oral Surgery, Faculty of Medical Sciences in Zabrze, Medical University of Silesia in Katowice, Pl. Akademicki 17, 41-902 Bytom, Poland (tmorawiec@sum.edu.pl).

## Sample Availability

Samples of the compounds aren’t available from the authors.

## Abbreviations

TTO	Tea tree oil
EEP	Ethanolic extract of propolis
API	Approximal Plaque Index
mSBI	modified Sulcus Bleeding Index
OHI-s	simplified Oral Hygiene Index
DPI	Denture Plaque Index
